# The Particle of Haag’s Local Quantum Physics: A Critical Assessment

**DOI:** 10.3390/e26090748

**Published:** 2024-09-01

**Authors:** Gregg Jaeger

**Affiliations:** Quantum Communication and Measurement Laboratory, Department of Electrical and Computer Engineering, and Division of Natural Science and Mathematics, Boston University, Boston, MA 02215, USA; jaeger@bu.edu

**Keywords:** elementary particle, quantum field theory, quantum event, local quantum physics, ontology

## Abstract

Rudolf Haag’s Local Quantum Physics (LQP) is an alternative framework to conventional relativistic quantum field theory for combining special relativity and quantum theory based on first principles, making it of great interest for the purposes of conceptual analysis despite currently being relatively limited as a tool for making experimental predictions. In LQP, the elementary particles are defined as species of causal link between interaction events, together with which they comprise its most fundamental entities. This notion of particle has yet to be independently assessed as such. Here, it is captured via a set of propositions specifying particle characteristics and then compared to previous particle notions. Haag’s particle differs decisively with respect to mechanical intuitions about particles by lacking, among other things, even an approximate independent space–time location. This notion is thus found to differ greatly even from those of relativistic quantum mechanics and quantum field theory, which have been applied to the known elementary particles.

## 1. Introduction

The role, if any, of the particle in the fundamental ontology of subatomic physics has long been the subject of controversy. Recently, there has been a stalemate in the debate between positions favoring either particles or fields as the fundamental objects, especially after it was shown that neither alternative is definitively superior [1]. Nonetheless, the tendency in the investigation of the foundations of physics has been to view the field as at least more fundamental than the particle. And, most often, the field has been taken to be the *only* fundamental sort of physical entity (cf. [2]) despite constant apparent reference to elementary particles in scientific practice, in particle physics (PP) in particular. The ‘third alternative’—including Rudolf Haag’s position—which considers *physical events* to be fundamental, is less often considered, despite the influence of Bohr’s views (cf. [3], p. 109) which considers events to be of primary significance. Haag’s Local Quantum Physics (LQP) considers physics as taking place via causal links between these local events, some of which are identified as elementary particles [4,5,6]. Haag’s particle notion differs greatly in character not only from those of classical mechanics (CM) and quantum mechanics (QM), but also from that of conventional quantum field theory (CQFT), the basic theory applied in the practice of PP. Haag takes events and their causes, including elementary particles, as the fundamental physical elements of reality that form an independent causal network and constitute the universe [7]. (They are also taken to ground space–time, although he does not address the general-relativistic case; cf. [6]). The successive local causal realization of events involving particles and other, more complex objects is taken to provide a fundamental direction of time. The particle is just the simplest sort of causal link in LQP, possessing only ‘structural’ properties, including rest mass, spin, and charge, that comes to be reified with the causal pairing of sequential, distinct physical events. (Rather than being relational as structural properties are often understood, for Haag, these properties are just those intrinsic to it, being symmetry invariant). The equations of motion of LQP theory thus govern the probabilities of the occurrence of real events but not any motion of particles through space–time.

The nature of particles in LQP differs from that of standard notions. That nature is spelled out in detail here via nine propositions, which aid in the assessment of its adequacy based on the characteristics they specify; it is not the purpose of this article to extend Haag’s theory of relativistic quantum physics but rather to make explicit the distinct character of particles within it. I argue that, despite lacking several traditional corpuscular characteristics and being in some ways radically different from any previous quantum particle, this entity can still be considered a sort of particle related to previous notions. Here, first, Haag’s LQP is outlined in Section 2 for context; the limited sense in which causal links between events are analyzable in that framework is explicated in Section 3, where the formal treatment of interactions in LQP (that is, his events) is also summarized; and the characteristics attributed to the particle by Haag are given in Section 4 as a set of propositions. Finally, Haag’s particle notion is critically assessed in Section 5.

## 2. Grounding Relativistic Particles: Theoretical Context

Although various forms of special-relativistic quantum theory have been used to conceive the experimentally recognized elementary particles, each takes them to relate differently to the theories’ fundamental ontologies. Historically, the difficulties in describing the atomic and subatomic phenomena in terms of the commonsensical classical notion of particle (and/or field) became clear in the investigation of phenomena such as the energies appearing in the emission and absorption spectra of hydrogen, first adequately explained via QM, which is the physics of quantum systems with finite numbers of degrees of freedom that takes over the traditional mechanical vocabulary and adapts it to the atomic and subatomic realm. This adaptation already involves a non-corpuscular notion of particle, in that it could also exhibit apparently wave-like behavior with no underlying medium. And it is also already evident there that individual objects of a mechanical sort are well defined only under appropriate circumstances (cf. [8]). System isolation is almost always only approximate because interaction entangles states, and localization is at best temporary (even non-relativistically; cf. [9], p. 24) because, among other behaviors, system states generally disperse spatially. Quantum state entanglement brings the very individuality of quantum particles into question when states are interpreted as objective system states. And the lone traditional particle can be captured in the quantum world only approximately and in the non-relativistic limit, where its temporary localization can be well defined. Moreover, in (relativistic) QFT, where quantum fields have an infinite number of degrees of freedom, no robust sense of particle localization is available (cf. [10]).

A standard approach in quantum theory is to quantize a classical field using appropriate operators on a Hilbert space of states which provides a quantum field with the useful feature of spatiotemporal continuity as in CQFT. The fields are then the dynamical variables, and special relativity is incorporated by requiring covariance under the Lorentz group of space–time transformations. Relativistic particles are then standardly defined as excitations of the positive and negative mode frequencies of the free fields. These excitations naturally correspond to a discrete difference in field energy with a corresponding momentum and can be counted in the interaction-free setting via an appropriate excitation number operator *N* on the corresponding Fock–Hilbert space F that naturally enforces the appropriate multi-particle statistics. Each sort of such particle then appears straightforwardly to have a corresponding field of which it is an aspect. These fields along with space–time are standardly taken as the fundamental objects of the theory’s ontology; particles are considered in conventional free QFT to reduce ontologically to fields in which they propagate freely and are, therefore, considered non-fundamental.

However, incorporating quantum particles via fields is far less straightforward in a theory that includes *interactions* in addition to simple propagation. The particle is no longer similarly definable except in the free field limit of large times after interaction, that is, when an asymptotic condition is satisfied [2]. (Furthermore, there exists no unitary transformation from the corresponding pre-interaction states to the post-interaction states within a single Hilbert state space as seen in the well-known theorem bearing Haag’s name). Particle interaction processes cannot be readily accounted for in practice within a relativistic QFT without, at least, resorting to a perturbation-theoretical approach, and even then, the eigenvalue of *N* is generally indefinite. (For an outline of one manner of consistently defining particles in the context of quantum perturbation theory, via the LSZ formalism, under a minimalist conception of particle according to Wigner’s method, see [11,12]). Thus, in CQFT, particles are typically considered transient field aspects or epiphenomena.

CQFT has made the most successful predictions in practice for what is observed in PP, even though many argue that, due to the eclectic character of its mathematical methods, it is not sufficiently well grounded in relativistic quantum physical principle. LQP attempts to ground relativistic quantum theory in a way that avoids these and other difficulties, such as infinite values for physical magnitudes, by demoting the role of quantum fields. In LQP, the LSZ formalism can still be used to predict particle-scattering event statistics at the subatomic scale, and matter still appears quasi-mereologically structured: when particles are stable and can be related to quasi-local fields, it also allows for the description of the scattering of composite particle systems; cf. [13]. (How this is performed is sketched in the next section to provide mathematical context for the characterization and assessment of Haag’s particle notion later; a full summary of the basic structure of the LQP framework is given in [5], Chs. III–IV). Despite their demotion, quantum fields are still described in LQP. Particles, too, can be viewed in the very general mathematical setting of algebraic quantum field theory (AQFT) without requiring the asymptotic condition for their definition.

Use of the AQFT formalism has long been considered a means for deriving deeper insight into the broad implications of the foundational principles of relativity and quantum theory. The LQP approach within AQFT imposes the following requirements [13].

The stable **single-particle energies are positive**:

(**S**) The spectrum of the energy–momentum operators $P_{\mu }$ in

the Hilbert space H of quantum states is restricted to the closed

forward light cone ${\bar{V}}_{+}=\{p:p_{0}\geq \left|p|\right\}$.

It is also assumed that there is a unique ground state Ω, **the vacuum**. Only a finite number of covariant fields having a finite number of components are considered dynamical variables; finally, we have a principle of **locality**, (either)

(**L**) Field quantities that are space-like to each other, that either commute

or anti-commute

(or) its generalization, L_0_, given below. Given the requirements S and L, and quasi-local operators connecting Ω to states having a discrete energy spectrum, Haag argues that individual particles can be described in LQP (cf. [14]) in the limited circumstances discussed in the next section.

Now, one of the strongest differences of Haag’s LQP from CQFT regards space–time, which in LQP is reduced to the ordered structure of the network of physical events and the causal links between them. In CQFT (as in CM, QM), space–time is assumed to be an arena in which events take place that is given a priori, and its points serve as indices for the property values of fundamental quantities. (The structure of this causally ordered network as well as its relation to scattering is formalized in the next section). Because of its use of the mathematics of AQFT, LQP is often viewed as simply another form of field-based theory. However, it also introduces structures more complex and fundamental than fields, namely, nets N of algebras of observables. These are introduced in order to ensure the finiteness of the physical magnitudes.

When implementing the above principles in LQP, quantum fields are defined in terms of the N on local regions instead of being indexed by independent space–time points. The localized (Minkowski) space–time (M) regions O arise along with, and depend on the events of LQP. These quantum fields associate to each open region O in space–time an algebra R(O) of operators on Hilbert space H, namely, the algebra generated by all *smeared-out* fields Φ(f), where *f* is a test function having support in O. (It should be noted here that smeared-out fields appear necessary for providing a rigorously defined field basis in QFT, generally [1]). The correspondence O⟶R(O) connects the space–time regions with observables and is taken to satisfy three requirements:

*Isotony*: $O_{1}\subset O_{2}$ implies $R{\left(O\right)}_{1}\subset R{\left(O\right)}_{2};$

*Locality* $L_{0}$: Observables for space-like separated regions commute;

*Covariant action of the Poincaré group on the local algebras*.

Here, $L_{0}$ is a corresponding version of the locality principle *L* [13]. The elements of the algebra R(O) describe the familiar physical transformations in O, for example, energy annihilation/creation. N is thus the basis of the physical characterization of particle scattering processes in LQP. Quantum fields therefore serve to provide a coordination of this net of operator algebras (which is possible via a mapping from space–time coordinates to field values [4]).

This concludes our brief review of the broad theoretical background for considering Haag’s particle notion, which is applicable only under the special circumstances identified in the next section, which addresses interactions. (Note, again, that Haag’s particle concept was defined only for the special-relativistic context; cf. the requirement $L_{0}$. There is no pretense that Haag’s particle as presented is a general-relativistic particle).

## 3. Interaction Events in Local Quantum Physics

The events of LQP are understood as interactions caused via elementary particles and other, larger entities. In Haag’s theoretical framework, particles are defined primarily as directed links in a causal network as explicated in detail in the next section. He explains what he means by a local event as follows. “For many purposes it suffices to understand by the term ‘event’ just a detector signal. But …one can push this to finer distinctions: Invisible processes like microscopic triggering events or elementary reactions …which are reconstructed from many secondary detector signals” [15], p. 233. Haag argues that this is not problematic for the physical picture of LQP, and the objective reality of the past does not depend on any observation: some events correspond to the operation of designed and monitored detectors, while others happen ‘in the wild’, leaving traces that go unnoticed. “The simplest type of event is a collision process between particles, well isolated from other matter and closed by the spatial separation of the reaction products” ([7], p. 735).

Understanding how Haag’s particle is conceptualized beyond broad statements requires us first also to better formalize how interactions are represented in LQP because it is through them that they arise. The basis for the causal relationship between pairs of events in LQP is their time-like sequential occurrence. Haag can be viewed as invoking an adapted form Humean causation applicable in the quantum context: causes are events that appear prior to their effects in a regular way in sequence, with source events always appearing before target events and the directed causal links between them exactly conserving the particles’ intrinsic properties. Although quantum theory, in general, does not involve Hume’s first condition (spatiotemporal contiguity) in its original form (because trajectories are uncertain at best), Haag replaces the space–time contiguity with the ordered sequencing of events available to his framework. Hume’s second condition (a cause being prior to its effect) is reflected in his theoretical framework by the directed nature of his time-like progressing sequences. Hume’s third condition (constant union between cause and effect) is reflected by Haag’s requirement that there is no causal link (a particle) unless there is a target event (effect) after any source event; Hume, of course, also has much more complex events in mind in his discussion of causation than basic physical ones that are Haag’s effects (“target events”). For Haag, both an effect and its cause are events with physical traces, and Hume’s sameness requirement is that event pairing involves the conservation of (what he calls “structural”) properties such as spin and charge; this is always enforced and never violated so that no effect arises without the causal link bearing a correct measure of these properties.

Event sequences, rather than directed lines in a space–time diagram, are superior for their representation for reasons similar to those arising if one is tempted to consider Feynman diagrams as precise spatiotemporal illustrations of interactions in space–time, which they are not. The issue is more acute here because, in LQP, space–time itself is taken to depend on the regions associated with interaction events themselves. Each causal link of the network is directed from its “source” event (cause) to the succeeding “target” event (effect), which may, in turn, be the cause of another subsequent event, and so on. Events are partially ordered via causal efficacy: An event γ is later than α if α precedes it in a causal sequence, that is, if (…, α, …, γ, …). A causal boundary is indicated by this ongoing ordering: To each set of distinct source events $\left\{\alpha_{i}\right\}$—of distinct sequences, each corresponding to an value of *i*—there corresponds a set of “arrows”, $\left\{A_{\alpha_{i},\beta_{j}}\right\}$, ‘directed’ from each $\alpha_{i}$ toward its possible direct successors $\left\{\beta_{j}\right\}$ (j≥i) for its sequence. The **boundary** *S* (of ‘current’ time) is indicated by the set of ‘arrows’ $A_{\alpha_{i},\beta_{j}}$ for any set of events $\left\{\alpha_{i}\right\}$ in which the (most recent event) $\alpha_{i}$ (and no real successor $\beta_{i}$) has (yet) been realized. [Thus, the $\alpha_{i}$ are the relatively last events of their respective (sub-)sequences (which, at that point in history, are just $\left(\ldots ,\;\alpha_{i}\right)$) which, nonetheless, could still continue subsequently to the $\alpha_{i}$. Note also that these sequences can—and some do—intersect with each other].

Thus, there are two grand sets of events: $F_{S}$, those in the *future* relative to any boundary *S*, and $P_{S}$, those in the *past* relative to *S*. Haag considers the cutting of *S* at the (momentary) end of all (still developing) sequences of actualized events to be analogous to the cut (as a space-like surface) across the possible indeterminate trajectories of quantum systems within a traditionally continuous space–time diagram that lead into the future. No event in $P_{S}$ is later than any point in $F_{S}$, and $P_{S}$ contains no points coinciding with the boundary. That is, the ‘current’ boundary is indicated exactly by the truncation of each and every sequence at the relative ‘causal present’, which is indicated in each sequence $\left(\ldots ,\;\alpha_{i}\right)$ by its (currently) last term (already realized) and the arrows $\left\{A_{\alpha_{i},\beta_{j}}\right\}$ leading away from it.

Haag argues that the existence of local collision centers in regions at which particles (as defined by LQP) are realized (with the appearance of the target event of that center) is empirically evident, for example, by the tracks they leave in various sorts of detector in situations where the momenta of incoming matter are extremely precisely concentrated ([7], Sect. 3). The interaction length is taken to provide event localization in that case; cf. [7], Sect. 3. In the regime where events are sparse, an event is considered a particle collision process, and a number of causal sequences will coincide at the same event β. Haag holds that such scattering events are among those in which the particle notion applies. These events will be very diffuse when low energies are involved but sharp when energy–momentum transfer is high ([9], p. 29). Spatiotemporal locations of scattering processes under such conditions are those indicated at the cruxes of tracks in bubble-chamber photographs or reconstructions of interactions based on data provided by various particle-detection systems in experimental PP.

To find quantum probabilities for particle scattering, Haag considers an overall process, wherein *n* particles are present (due to prior events) associated with events with centers of mass $x_{i}$ (i=l,…,n), *and* detection events occur at much later times, in the neighborhood of the points $y_{k}$ (k=1,…,m). In quantum theory, the associated probability amplitude is $A=\sum A_{G}$. So each summand $A_{G}$ corresponds to a graph with external vertices $x_{i},y_{k}$, internal vertices $z_{l}$ (l=1,…,l), and (at most) one line is taken to connect each pair of vertices. (To visualize this, one can imagine combining generic topological diagrams similar to tree-level Feynman graphs).

Haag then holds that, for points $z_{l}^{\alpha }$ (α=1,…,f), one may take a vertex, connected by *f* lines, to be represented by the function $\Gamma (z_{l}^{1},\ldots z_{l}^{f})$, having a short extension, that depends only on the difference $z_{l}^{\alpha }-z_{l}^{\beta }$; the line from $z_{l}^{\alpha }$ to $z_{l^{\prime }}^{\alpha^{\prime }}$ will have a corresponding propagator, $\Delta (z_{l}^{\alpha }-z_{l^{\prime }}^{\alpha^{\prime }})$. Then, $A_{G}$ is the integral over the $z_{l}^{\alpha }$s of the product of the $\Gamma (z_{l}^{1},\ldots z_{l}^{f})$ and the $\Delta (z_{l}^{\alpha }-z_{l^{\prime }}^{\alpha^{\prime }})$; cf. [16,17,18]. (A two-dimensional illustration of a relatively simple particle-scattering interaction and representation of the detail of one relevant vertex can be found in [19], p. 249. Figures 1 and 2).

Then, in the limit of large time-like separations, the propagators Δ (associated with uncertain mass) go to functions $\Delta_{+}$ associated with particles of *definite mass*, that is, stable particles. Fourier decomposing the vertex representative function, Γ, and propagator $\Delta_{+}$, and evaluating the probability amplitude integral for $A_{G}$ for each line, Haag arrives at a momentum $p=m\left(z_{l}-z_{l^{\prime }}\right){\left({\left(z_{l}-z_{l^{\prime }}\right)}^{2}\right)}^{-1/2}$, where *m* is the corresponding mass value and $z_{l},z_{l^{\prime }}$ are mean values of the $z_{l}-z_{l^{\prime }}$ (cf. [19], p. 49); units here are taken such that *c* is unity as is standard in PP. As in calculations using the Feynman diagrammatic calculus, the total momentum must be conserved at each vertex. The space–time locations of the internal vertices are determined by the external points and the particle masses. The probability for the overall processes involving the event vertices is then taken to be

(1) $${\left|A\right|}^{2}=\sum {\left|A_{G}\right|}^{2}\;,$$

which Haag justifies, crucially, by requiring that the space–time distances between vertices be large enough that different graphs will have sufficiently different configurations of internal vertices for the interference terms to be negligible, and that only small numbers of graphs contribute to ${\left|A\right|}^{2}$. The sparseness condition invoked by Haag thus allows particles to be distinguished.

With these formalities in place, the calculation of the probabilities for the realization of specific causal patterns of particle collision mirrors the standard calculation procedure ([7], p. 5). To calculate these probabilities, Haag attributes each sort of causal link α a Hilbert space $H_{\alpha }$, considered as a subspace of the Fock space generated from the Hilbert spaces corresponding to all types of potential link. Events that are maximal in the sense of corresponding to the greatest state filters, that is, corresponding to the most precise measurement of the pertinent observables, fully specify the links leading to them in the spatiotemporal context. They will also select a product vector of factors for each type of link, including particles, e.g., $\phi_{\alpha }⊗\phi_{\beta }\in H_{\alpha }⊗H_{\beta }$, providing a state with possible superposition states in, e.g., $H_{\gamma }⊗H_{\delta }$. Probabilities for a subsequent event might, in turn, correspond to another such product state, if the event involves a sufficiently strong such state filter. (An illustration of such a situation can be found in [5], p. 314–315 with Fig. VII.3.1).

Under low event-density conditions, the target event β caused by local interactions involving such particles is described by Haag via a quasi-projection $I_{x}$ (of a reducible representation given by the tensor product of the irreducible representations associated with all the causal links leading to it) acting on the joint state–vector $\Psi^{\mathrm{in}}$ ([7], pp. 739–741). In order to compute the probabilities of a such scattering events, Haag finds the output probability amplitude for the event $\Psi^{\mathrm{out}}=\int I_{x}\Psi^{\mathrm{in}}d^{4}x$. To define the space–time region of an event, say, involving just two particles that can be well localized due to the interaction strength, he argues that the $I_{x}$ provide localization of the scattering amplitudes to roughly the interaction range. He then sharpens this localization using a cellular division in spatial coordinates by going from the standard continuous sums to discrete sums, enabling sharper localization:

(2) $$\int I_{x}dx=\sum I_{k};\;I_{k}=\int I_{x}g_{k}\left(x\right)dx;\;\sum g_{k}\left(x\right)=1;\;\Psi_{k}=I_{k}\Psi^{\mathrm{in}}\;,$$

where the functions $g_{k}$ have support in cells *k* chosen such that the individual amplitude terms $\Psi_{k}$ correspond to incoherent alternatives so that quantum interference effects that might preclude a clear particle interpretation do not appear. Among the alternative processes, just one is assumed to be actualized in the interaction event ([5], p. 318). In this way, particle interaction events are described, and scattering probabilities are provided in LQP as similarly as possible to standard methods.

## 4. The Particle of Haag’s LQP

Let us now consider the particle that Haag argues in broad terms appears in his treatment of local quantum interactions in the framework of LQP, given that it functions largely at the level of principle rather than within a specific theory. (See the introductory section of [20] for a valuable commentary on differences in the applicability of LQP vs. conventional quantum field theory). To aid the critical assessment of this notion, I first offer here the following list of short propositions capturing it, based on the descriptions in his foundational publications. (See [6] for an overview of Haag’s work on the foundations of quantum theory subsequent to the first appearance of his magnum opus, *Local Quantum Physics*). This section consists only of this list, which discusses the causal network of events as formalized above. The list is provided primarily for subsequent use with little narrative interlude beyond relevant quotes and citation(s) and brief contextual comments for each proposition. The propositions are arranged in an order that allows for clear reference of some propositions by others. These allow us, in the next section, to critically assess Haag’s particle concept in relation to previous particle notions, and mitigate the need to explicitly bring in the level of detail provided above.

(I) The particle is a causal link between two events.

[“A [causal] link is a messenger connecting a source event with a target event. In the simplest case (low-density situation), links correspond to the aforementioned objects (stable particles)”, [9], p. 28. This sense of ‘messenger’ particle is not that of exchange forces, cf. [21], because there, the term refers to *virtual* particles, which Proposition IX, stated below, explicitly precludes].

(II) A particle exists only if a corresponding target event occurs.

[A particle, a species of causal “link becomes established (an ‘element of reality’, if one wishes) only when the target event is concluded” [9], p. 28. This can be seen to satisfy something like Popper’s reality criterion for a putative entity, i.e., that it is real if “it can be kicked, and it can kick back”, in that the first event, the particle begins as a kick at one event and it ends as a kick with its later, target event].

(III) An ‘arrow’ without a target event does not represent a particle.

[“Potential links belong to the realm of possibilities, not facts. This illustrates the sense in which we can speak of individual objects”; [9], p. 28. Thus, “we can speak of the individual electron which was ejected from a metal surface by a radiation pulse and subsequently caused a click in a detector. But it makes no sense to talk about an individual electron without reference to specific events”; [9], p. 28. For a discussion of the notion of potentiality, cf., e.g., [22]].

(IV) The particle has a unity indicating its individuality in that

it excites *only one* detector.

[Haag calls this unity a “staying together”, corresponding to being “permanently singly localized” in this sense. For elementary particles, this corresponds to sharp mass value [15], p. 222. In LQP, a ‘detector’ is a portion of the environment and subsumes (at least parts of) the detection apparatus (the “prototypes of observables”, [9], p. 37) but is *not restricted to purpose-built apparatuses*. Detection is, accordingly, not measurement in the narrow sense of necessarily involving a consciously monitored apparatus triggering process but an aspect of the interaction process in which a target event appears].

(V) The particle is well defined only if the two events it links

satisfy the sparsity approximation.

[The particle is “an asymptotic notion which can be arbitrarily well approximated by isolation” of the events it causally relates in their surroundings; [7], p. 735. This sparsity approximation is that in which the simplest type of event takes place: the particle collision as discussed in Section 3, above].

(VI) The particle has only purely structural attributes, comprising only

mass, charges, spin–magnitude, and possibly others related to the *internal*

structure.

[“We know empirically that stable particles can be isolated and that each type has a specific internal structure which can be regarded as a true attribute, an ‘element of reality’”; [4], p. 310. For Haag, there are two levels of ‘structure’ regarding particles, the internal structure, which is simply the collection of its intrinsic properties mentioned in VI, and external structure, which regards its relationship to events and, by extension, their location in the causal network].

(VII) The particle has no quantum state; particle-indexed

probability amplitudes do not refer to particles themselves.

[For those events consisting in the uniformly (un)directed emission of a particle, any probability amplitude (‘state’) $\Psi^{\mathrm{in}}$ refers only to a corresponding space–time location of the center of a second, target *event* of the pair that the particle causally links to that preceding, source event: a “‘state’ is a propensity assignment relevant for the occur[re]nce of a subsequent event”; [23], p. 63. And, “In general the source event does not even determine the nature of the potential links originating from it”; [9], p. 28. So, if entanglement is a characteristic of *states* as it is standardly defined, then LQP particles themselves, unlike Hilbert-space vectors, cannot be entangled].

(VIII) The values of those spatiotemporal quantities measured

during an interaction are not attributes of particles themselves

but regard that event of interaction.

[Haag views this as in accord with the motto “‘a particle does not have a position at any given time unless it is measured’. In our words: a causal link does not have space–time attributes (apart from those due to the events it connects)…” ([9], p. 28). “…after the source event of emission of a particle we represent it by a roughly spherical wave function. This should not be interpreted as relating to the probability for the changing position of a point-like particle but rather to the probability for the space–time location of the collision center in a subsequent event” [5], p. 313. Rather than being considered intrinsic, properties measured during an event of interaction, for example, with a purpose-built external apparatus, regard the event of interaction specifically. (See [24] for an analogous discussion of the pertinence of measurement data to states in that case)].

(IX) There are no virtual particles.

[Virtual ‘particles’ have no target event as defined in LQP; they leave no trace corresponding to ‘detection’ and are represented only as arrows, not as causal links. By IV, they could have no unity or individuality in Haag’s sense. See [7], p. 735. “The simplest type of event is a collision process between particles, well isolated from other matter and closed by the spatial separation of the reaction products. Its mental decomposition into subevents or ‘virtual events’ (as in Quantum Field Theory) is a useful model but no individual existence of the virtual events can be claimed” [7], p. 735].

## 5. The Innovations of Haag’s Particle Notion

With the essential background of LQP provided and the causal network now formalized, and the characteristics of Haag’s particle stated more straightforwardly in the last section, I now assess it as a particle in comparison first to a general notion of particle, and then to others that have been used in quantum theory. Through this comparison, one finds that Haag’s particle deviates significantly from intuitive understandings of what a particle should be like.

As a first step in the assessment of this notion of particle, let us compare its characteristics with the general characteristics of the particles that have heretofore been accepted in physics (from at least the emergence of classical physics onward). Let us to do so by reference to the list of particle characteristics provided by Brigitte Falkenburg [25] (after its critical reconsideration by [26]). Falkenburg’s analysis concludes that particles are as follows:

(PROP) have some intrinsic properties;
(INDEP) can be independent of each other;
(POINT) are point-like in interactions;
(CONS) obey conservation laws;
(LOCD) are localizable by a detector;
(DISC) are discontinuous [i.e., they come in quanta (of matter or radiation)].
One sees from Propositions I–IX in the context of LQP that Haag’s particle can be considered a particle because it meets these requirements, with the strong caveat that it satisfies them quite differently in both manner and degree from previous quantum theoretical notions, particularly those of CQFT.

The greatest difference is found in the way that DISC is satisfied: Although Haag’s particles are countable and, therefore, are quanta only in the general sense of satisfying DISC most literally, they are not understood to be so by being excitations of fields *per se*: they have only the defining invariant characteristics recognized by particle physics and bear units of those characteristics. The Haag particle is *not* defined by direct reference to field quantities, nor is it a particle in the sense of Bohmian mechanics, which possess definite trajectories. Haag particles also differ in the manner that they can be said to satisfy POINT, in that in LQP space–time, points themselves depend on local events.

Now, let us consider the character of Haag’s particle both in relation to the particles of QM and CQFT, with specific reference to each of Propositions I–IX in succession, as well as in relation to the characteristics attributed to particles in the ontologies within the classical physical framework, keeping in mind that elementary particles have long been considered not to be classical-type particles (with the exception, perhaps, of those in Bohmian mechanics) as discussed in the Introduction.

Propositions I–III, defining the particle as a causal link between two events α and β that is real only if the second (effect) event, β, occurs, imply that a particle also exists only for a finite duration. The latter characteristic runs contrary to the ancient-to-classical view of the particle as eternal, not included in the above list of characteristics due to its rejection in quantum theory in general. Haag himself points out that the elements of his ontology differ from Maxwell’s view of elementary objects, namely, a universe of atoms wherein “the foundation stones of the material universe remain unbroken and unworn. They continue this day as they were created—perfect in number and measure and weight” (J. C. Maxwell, cf. [3], p. 298). (However, this finiteness is not novel: all relativistic field theories recognize that particles can be created and destroyed, e.g., by particle–antiparticle annihilation).

This also contradicts the (classical) view of particles as re-identifiable because they are identified only once (by the two events they link). But, again, the constraints of quantum multi-particle statistics already strongly suggest that particle re-identifiability is untenable as an absolute requirement because the identifiability of one particle among others is impossible in the absence of it being singled out at detection; cf. [27]. Because re-identifiability also does not appear in the list of general particle characteristics above, this does not disqualify Haag’s putative particle as a sort of particle.

Proposition IV, explicating what is essential to particle *individuality*, then differentiates Haag’s approach to particles from that of standard QM and CQFT by connecting its unity directly to the appearance of events. The detection event is Haag’s archetypal event, and his explication of particle identity (‘unity’) is a “staying together” by virtue of an association with only *one* target event after the source event. It is not identified by a particular physical state ψ *per se* or any associated field being in such a state; according to Haag, ψ characterizes only a possible future event, not any *system*. The phrase ‘permanently singly localized’ can only refer to its target event (see Haag’s comment below under Proposition VIII), which accords with the general particle characteristic LOCD in that qualified sense. It is standardly assumed in physics that a particle can cause a future event, and even in QM, ψ is understood to provide (at a minimum) knowledge of a future event (involving the system ψ is attributed to).

Proposition V specifies Haag’s fundamental requirement that there be a target event β for each predecessor event α in order for there to be a particle cause at all. It is self-evident that there must be an effect for causation to take place. So it is in itself unproblematic for his concept of particle.

Proposition VI, which specifies the characteristics attributable to a particle, namely, mass, charges, spin–magnitude (and possibly others having to do with further internal characteristic properties) is less than what is classically considered attributable to a particle; indeed, it does *not* include the ancient-to-classical view that it always (‘permanently’) has a space–time location along a trajectory. But, it does accord with the minimal set of recognized elementary particle attributes (which Haag calls ‘structural’, recognized by PP) that Max Born has taken as sufficient for their indication: “The main invariants are called charge, mass (or rather: rest mass), spin, etc.; and in every instance, when we are able to determine these quantities, we decide we have to do with a definite particle. I maintain that we are justified in regarding these particles as real in a sense not essentially different from the usual meaning of the word” [28]. However, it greatly limits the physical significance of Haag’s particle.

Proposition VII reinforces the limitations on the properties specified by Proposition VI by explicitly excluding the standard attribution of dynamical properties to particles, e.g., by ψ via the eigenvalue–eigenstate link.

Proposition VIII indicates that the general requirements POINT, CONS, and LOCD cannot hold for the Haag particle alone but instead hold *only* for the particle–event complex. This, together with its being primarily a causal link rather than a substance (in the ontological sense), strongly differentiates Haag’s particle notion from those coming before it, and takes the particle notion to a new level of austerity. With this, Haag particle notion breaks with all previous mechanistic intuition regarding particles and is bound existentially to events.

Proposition IX accords with the standard view of virtual particles found in QM and QFT, which is that they have no actual reality. Considering them may have a heuristic use—as Haag commented as shown in the previous section regarding this proposition—but they have no ontological significance in LQP.

With these observations, we find that Haag’s definition of particle alters the significance of specific results of interest in the foundations of quantum theory if it is adopted. For example, non-classical correlations are observed in distantly performed, joint measurements of spin-angular momentum components. There, the probabilities of patterns of future events required do not factor, generally, in that the corresponding probability amplitudes for these events can be entangled, where the state is (sub-)system indexed. In the standard parlance of quantum theory, it is often said that particle *subsystems* are entangled. However, in LQP, *there are also no such individual particles* to be considered (until both target events have occurred). LQP does not consider particles to be self-sustaining, variable quantum-state bearing systems. Therefore, associated Bell-type inequality violations have no implication for Haag particles but only indicate the failure of classical assumptions about the strength of correlations between distant *events* having more complex causes. This resolution comes at the price of rejecting much of what a particle has been considered to be in the history of physics and also precludes an explanation of such coincidence events by reference to them.

## 6. Conclusions

Haag’s particle is presented as a fundamental entity—an ‘element of reality’—captured here by Propositions I–IX, with instances lying within the fundamental causal network of events, formalized here, making up the fundamental reality according to LQP. This austere entity appears in only specific, but empirically significant, broadly occurring situations such as scattering processes in particle physics. It is also required to be stable and non-virtual. Beyond the general characteristics of independence, discontinuity, interacting in a point-like manner (remembering that in LQP space–time points themselves depend on interaction), and local detectability, which have long been attributed to particles in some fashion, Haag’s particle has only causal efficacy and the structural, special-relativistically invariant attributes of (rest) mass, charge, and total intrinsic angular momentum (and possibly other internal structural characteristics). Haag’s characterization is, therefore, only consistent with a very broad interpretation of the general criteria for an entity to be considered a particle. It excludes the intuitive corpuscular characteristics already shunned in most of special-relativistic quantum theory, but it *also* excludes all non-structural, dynamical properties. For this reason, Haag’s notion of particle could be seen as unacceptably weak; Haag’s particle bears very little resemblance to previous conceptions of physical objects, particularly those described directly by equations of motion. However, it serves to capture uniquely the scattering processes of high-energy physics as objective occurrences.

## Data Availability

The original contributions presented in the study are included in the article, further inquiries can be directed to the corresponding author.
